# Predictors and Outcomes of Neurological Deterioration in Intracerebral Hemorrhage: Results from the TICH-2 Randomized Controlled Trial

**DOI:** 10.1007/s12975-020-00845-6

**Published:** 2020-09-09

**Authors:** Zhe Kang Law, Rob Dineen, Timothy J England, Lesley Cala, Amit K Mistri, Jason P Appleton, Serefnur Ozturk, Daniel Bereczki, Alfonso Ciccone, Philip M Bath, Nikola Sprigg, on behalf of TICH-2 investigators

**Affiliations:** 1 Stroke Trials Unit, Division of Clinical Neuroscience, University of Nottingham, City Hospital, Nottingham, NG5 1PB UK; 2 Department of Medicine, National University of Malaysia, Kuala Lumpur, Malaysia; 3 Radiological Sciences, Division of Clinical Neuroscience, University of Nottingham, Nottingham, UK; 4 NIHR Nottingham Biomedical Research Centre, University of Nottingham, Nottingham, UK; 5 Vascular Medicine, Division of Medical Sciences and GEM, University of Nottingham, Nottingham, UK; 6 School of Medicine, University of Western Australia, Perth, Australia; 7 Stroke Medicine, University Hospitals of Leicester NHS Trust, Leicester, UK; 8 Department of Neurology, Selcuk University Medical Faculty, Konya, Turkey; 9 Department of Neurology, Semmelweis University, Budapest, Hungary; 10 Neurology Unit, Azienda Socio Sanitaria Territoriale di Mantova, Mantua, Italy; 11 Department of Stroke, Nottingham University Hospitals NHS Trust, Nottingham, UK

**Keywords:** Neurological deterioration, Intracerebral hemorrhage, Tranexamic acid, Randomized controlled trial, Stroke, Hematoma expansion

## Abstract

Neurological deterioration is common after intracerebral hemorrhage (ICH). We aimed to identify the predictors and effects of neurological deterioration and whether tranexamic acid reduced the risk of neurological deterioration. Data from the Tranexamic acid in IntraCerebral Hemorrhage-2 (TICH-2) randomized controlled trial were analyzed. Neurological deterioration was defined as an increase in National Institutes of Health Stroke Scale (NIHSS) of ≥ 4 or a decline in Glasgow Coma Scale of ≥ 2. Neurological deterioration was considered to be early if it started ≤ 48 h and late if commenced between 48 h and 7 days after onset. Logistic regression was used to identify predictors and effects of neurological deterioration and the effect of tranexamic acid on neurological deterioration. Of 2325 patients, 735 (31.7%) had neurological deterioration: 590 (80.3%) occurred early and 145 (19.7%) late. Predictors of early neurological deterioration included recruitment from the UK, previous ICH, higher admission systolic blood pressure, higher NIHSS, shorter onset-to-CT time, larger baseline hematoma, intraventricular hemorrhage, subarachnoid extension and antiplatelet therapy. Older age, male sex, higher NIHSS, previous ICH and larger baseline hematoma predicted late neurological deterioration. Neurological deterioration was independently associated with a modified Rankin Scale of > 3 (aOR 4.98, 3.70–6.70; *p* < 0.001). Tranexamic acid reduced the risk of early (aOR 0.79, 0.63–0.99; *p* = 0.041) but not late neurological deterioration (aOR 0.76, 0.52–1.11; *p* = 0.15). Larger hematoma size, intraventricular and subarachnoid extension increased the risk of neurological deterioration. Neurological deterioration increased the risk of death and dependency at day 90. Tranexamic acid reduced the risk of early neurological deterioration and warrants further investigation in ICH. URL:https://www.isrctn.com Unique identifier: ISRCTN93732214

**Electronic supplementary material:**

The online version of this article (10.1007/s12975-020-00845-6) contains supplementary material, which is available to authorized users.

## Introduction

Neurological deterioration affects approximately one-third of patients with spontaneous intracerebral hemorrhage (ICH) and increases the risk of death and dependency [1–3]. Older age, prior use of anticoagulant, larger baseline hematoma, CT angiography spot sign, hematoma expansion, perihematomal edema, intraventricular hemorrhage, subarachnoid extension, hydrocephalus and leukoaraiosis were reported to increase the risk of neurological deterioration after ICH in previous studies [2–6]. Nevertheless, apart from hematoma expansion, reports of other neurological and systemic complications associated with neurological deterioration were not well documented [2–6].

Tranexamic acid is an antifibrinolytic agent that was effective in preventing death due to bleeding in major trauma and traumatic brain injury [7, 8]. In the Tranexamic acid in Intracerebral Hemorrhage-2 (TICH-2) trial, there was no significant difference in death and dependency at day 90 between ICH patients treated with tranexamic acid and placebo [9]. However, there was a significant reduction in the rates of hematoma expansion, early death and serious adverse events with tranexamic acid [9].

## Aims/Hypothesis

We aimed to examine the predictors of neurological deterioration in ICH and the effects on day 90 clinical outcome. We also explored the effect of tranexamic acid on neurological deterioration in the first seven days after ICH.

## Methods

The TICH-2 trial was a prospective multicentre randomized placebo-controlled trial testing the efficacy and safety of intravenous tranexamic acid in patients with acute spontaneous ICH presenting within eight hours of onset. Details of the trial have been published [9, 10]. Approvals were obtained from relevant national and institutional review boards prior to commencement. Written consent was obtained from the patients or their representatives prior to enrolment into the trial.

### Definition of Neurological Deterioration

Neurological deterioration was defined a priori as an increase in National Institutes of Health Stroke Scale (NIHSS) of ≥ 4 points or a decline in GCS of ≥ 2 points in our statistical analysis plan [10], as assessed at baseline (pre-randomization), day 2 and day 7. This definition with a cut-off of ≥ 4 points in NIHSS has a relatively high sensitivity and specificity for mortality (0.909 and 0.907) as compared with a lower NIHSS cut-off of ≥ 2 points (0.955, 0.685) [11]. In addition, clinical neurological deterioration was defined as that reported on the day 7 case report form, where investigators answered “yes” or “no” to whether the patient had an increase in NIHSS ≥ 4 or a decline in GCS ≥ 2 in the first 7 days, and specified the date it occurred. This reported neurological deterioration was irrespective of changes in NIHSS/GCS between the timed assessments (day 2 and day 7) because deterioration could also be transient [12], the participant may have recovered after treatment (e.g. neurosurgical intervention) or could not be assessed due to intubation. Neurological deterioration was considered early (END) if first occurred within the first 48 h, and late (LND) if first occurred between 48 h and 7 days [1, 2]. The time neurological deteriorations occurred was specified by the investigators in the day 7 form or serious adverse event (SAE) reports.

### Imaging Analysis

Measurements of hematoma, intraventricular hemorrhage and perihematomal edema (PHE) volumes were performed by three independent raters blinded to clinical data using semi-automated segmentation tools of ITK-SNAP software version 3.6.0 [13, 14]. Radiological adjudication including location of hematoma and presence of IVH was performed by a group of qualified neuroradiologists. Hematoma expansion was defined as an increase in intraparenchymal hematoma volume on follow-up scan (at 24 h) of > 33% or > 6 mL from baseline volume. Hematoma progression was defined as a composite of intraparenchymal, intraventricular or subarachnoid hemorrhagic expansion, or in case follow-up scans were not available, the occurrence of END or death before day-2 clinical assessment [15].

### Serious Adverse Events Reporting and Adjudication

Serious adverse events (SAE) were any events that were life-threatening, resulted in death, disability, hospitalization or prolongation of existing hospitalization or considered medically important. All SAEs were reported in the first 7 days, while fatal SAEs and safety outcomes (arterial and venous thrombosis and seizures) were reported up to 90 days. All SAEs including neurological deterioration were adjudicated by assessors who were consultant stroke physicians, based on clinical and diagnostic information provided by investigators.

### Statistical Analysis

Baseline characteristics of patients with END or LND were compared with those with no neurological deterioration. Multivariable binary logistic regression analyses were performed to determine the predictors of END and LND and their effect on outcomes. Variables that were significant on univariate analyses (*p* < 0.05) were included in the models. To determine the effects of tranexamic acid on END and LND, multivariable binary logistic regression analyses were performed with adjustment of covariates, as prespecified in the primary publication and statistical analysis plan with addition of baseline hematoma volume [9, 10]. These covariates were chosen as they were minimization factors and stratification used during randomization (age, sex, systolic blood pressure, NIHSS, onset-to-randomization time, prior antiplatelet therapy, country of recruitment and intraventricular hemorrhage). Baseline hematoma volume, though not available at randomization, was included as well, as it is a known key prognostic factor. *p* of < 0.05 was considered statistically significant and 95% confidence intervals (CI) are given. Analyses were performed using Statistical Package for the Social Sciences (SPSS) version 26 (IBM, Armonk, NY).

### Data Availability Statement

The trial data can be shared, upon reasonable request to the corresponding author and trial steering committee.

## Results

Of the 2325 patients recruited into the trial, data on neurological status was available in 2317 (99.7%). Seven hundred thirty-five patients (31.7%) had neurological deterioration within 7 days from onset of ICH. These include 590 patients with increase in NIHSS ≥ 4 or decline in GCS ≥ 2 (336 with an increase in NIHSS ≥ 4 only and 479 with decline in GCS ≥ 2 only; 225 with both an increase in NIHSS ≥ 4 and a decline in GCS ≥ 2). In 145 patients, the occurrence of neurological deterioration was based on the day 7 case report form, where investigators confirmed that there was a change in NIHSS or GCS fulfilling the definition but did not provide the actual scores. END and LND occurred in 590 (25.5%) and 145 (6.3%) respectively. The timings of neurological deterioration were recorded in 716 patients, including in all 590 with END (≤ 6 h 134, 5.8%; 6–24 h 282, 12.2%; 24–48 h 174, 7.5%) and 126/145 of LND. In 19 patients with LND, there was a change in NIHSS or GCS fulfilling the definition for neurological deterioration but the time the neurological deterioration occurred was not recorded.

Patients with END and LND were likely to be older, have previous ischemic stroke, intracerebral hemorrhage, ischemic heart disease, antiplatelet therapy, more severe stroke at onset (higher NIHSS, lower GCS) and a raised leukocyte count or blood glucose (Table 1). Patients with neurological deterioration were more likely to be issued with a “do not attempt resuscitation” (DNAR) order in the first 7 days (369, 50.2% vs 137, 8.8%; *p* < 0.001; Table 1). Of the 369 patients with neurological deterioration and DNAR order, in 114 (30.9%) a DNAR order was issued at least one day before neurological deterioration occurred while a DNAR order was issued on the same day in 167 (45.3%) or after neurological deterioration occurred in 82 (22.2%); the timing was missing in 6 (1.6%) patients. While the date of DNAR order issue was recorded, the time was not, so it is not clear whether DNAR was issued before or after neurological deterioration in patients who had DNAR issued on the same day as deterioration. The proportion of patients who had neurosurgery, invasive ventilation and intensive care unit admission was higher in patients with neurological deterioration (Table 1). There were higher proportions of death within 48 h in patients with END and within 7 days in patients with END and LND compared with patients with no neurological deterioration (Table 1). Notably, of 97 patients who died ≤ 48 h, 96 (99%) had END prior to death.

**Table 1:** Comparison of clinical characteristics by neurological deterioration status

Characteristics	Early neurological deterioration (*n* = 590)	No deterioration (*n* = 1582)	*p* value^a^	Late neurological deterioration (*n* = 145)	*p* value^b^
Age (years)	72.4 (13.4)	67.3 (13.7)	< 0.001	72.9 (12.2)	< 0.001
Sex (male)	300 (50.8)	904 (57.1)	0.009	92 (63.4)	0.14
Country,					
UK	543 (92.0)	1246 (78.8)	< 0.001	114 (78.6)	0.97
Non-UK	47 (8.0)	336 (21.2)		31 (21.4)	
Premorbid mRS (/5)	0 [0, 1]	0 [0, 0]	< 0.001	0 [0, 1]	0.097
Admission SBP (mmHg)	176.8 (30.9)	174.4 (29.6)	0.098	172.1 (27.5)	0.37
GCS (/15)	13 [11, 15]	15 [13, 15]	< 0.001	14 [11, 15]	< 0.001
NIHSS (/42)	18 [11, 22]	10 [5, 16]	< 0.001	17 [11.5, 20]	< 0.001
Antiplatelet therapy	216 (36.7)	343 (21.7)	< 0.001	50 (34.5)	< 0.001
Previous ischemic stroke/TIA	105 (18.1)	196 (12.4)	0.001	29 (20.9)	0.005
Previous ICH	52 (8.9)	61 (3.9)	< 0.001	12 (8.3)	0.011
Ischemic heart disease	68 (11.8)	118 (7.5)	0.002	17 (12.2)	0.047
Hypertension	361 (62.3)	965 (61.1)	0.60	90 (62.5)	0.75
Diabetes mellitus	80 (13.7)	204 (12.9)	0.63	26 (18.1)	0.082
Atrial fibrillation	16 (2.8)	47 (3.0)	0.79	8 (5.6)	0.081
Onset-to-CT time (hours)	2.1 (1.1)	2.3 (1.4)	0.001	2.4 (1.2)	0.52
Raised leukocyte count^c^	131 (22.9)	250 (16.1)	< 0.001	38 (26.6)	0.001
Temperature > 37.5 °C	14 (2.5)	43 (2.8)	0.72	2 (1.4)	0.35
Glucose (mmol/L)	7.5 (2.7)	7.1 (2.8)	0.012	7.6 (2.7)	0.050
Management within 7 days					
Tranexamic acid	283 (48.0)	807 (51.0)	0.21	66 (45.5)	0.20
Antihypertensive agent(s)	429 (73.6%)	1367 (86.6%)	< 0.001	121 (83.6%)	0.29
DNAR, day 2	251 (42.5%)	107 (6.8%)	< 0.001	31 (21.4%)	< 0.001
DNAR, day 7	316 (53.6%)	137 (8.8%)	< 0.001	53 (36.5%)	< 0.001
Neurosurgery	83 (14.1%)	24 (1.5%)	< 0.001	14 (9.7%)	< 0.001
Invasive ventilation	114 (19.5)	25 (1.6%)	< 0.001	27 (18.9%)	< 0.001
Intensive care	130 (22.1%)	67 (4.2%)	< 0.001	35 (24.1%)	< 0.001
Outcome					
Death ≤ 48 h	96 (16.3%)	1 (0.1%)	< 0.001	0 (0)	-
Death ≤ 7 days	196 (33.4%)	8 (0.5%)	< 0.001	18 (12.4%)	< 0.001

Data are number (%), median [interquartile range] or mean (standard deviation). Statistics are *t*-Student, Mann-Whitney *U* and chi-squared test: ^a^comparison of early neurological deterioration and no deterioration; ^b^comparison of late neurological deterioration and no deterioration. ^c^Leukocyte count of > 11.0 × 10^9^/L was considered to be raised. *CT*, computed tomography; *DNAR*, do not attempt resuscitation; *NIHSS*, National Institutes of Health Stroke Scale; *TIA*, transient ischemic attack

Both END and LND were associated with intraventricular hemorrhage, subarachnoid extension, lobar location, larger baseline hematoma and edema volume, midline shift and leukoaraiosis on CT (Table 2). Patients with END and LND had significantly more hematoma expansion and edema growth (Table 2).

**Table 2:** Comparison of radiological findings by neurological deterioration status

Radiological findings	Early neurological deterioration (*n* = 590)	No deterioration (*n* = 1582)	*p* value^a^	Late neurological deterioration (*n* = 145)	*p* value^b^
Baseline CT					
CT available	576 (97.6)	1550 (98.0)		139 (95.9)	
Lobar hematoma	260 (44.6)	368 (23.7)	< 0.001	55 (38.5)	< 0.001
IVH	272 (46.3)	367 (23.6)	< 0.001	67 (46.9)	< 0.001
Hematoma volume (mL)	42.8 (34.1)	15.7 (18.5)	< 0.001	38.3 (31.6)	< 0.001
IVH volume (mL)	5.2 (11.2)	2.1 (6.3)	< 0.001	6.7 (11.7)	< 0.001
Subarachnoid extension	150 (25.6)	123 (7.9)	< 0.001	25 (17.5)	< 0.001
PHE volume (mL)	21.8 (20.7)	9.1 (10.8)	< 0.001	18.7 (17.2)	< 0.001
Midline shift ≥ 5 mm	206 (35.8)	124 (8.0)	< 0.001	40 (28.8)	< 0.001
Leukoaraiosis	325 (55.5)	654 (41.9)	< 0.001	75 (52.4)	0.015
Old vascular lesion	346 (59.0)	951 (61.0)	0.42	93 (65.0)	0.34
Cerebral atrophy	530 (90.4)	1428 (91.5)	0.42	135 (94.4)	0.23
24-h CT					
CT available (baseline and 24 h)	457 (77.5)	1483 (93.7)		134 (92.4)	
Hematoma expansion	269 (58.9)	260 (17.5)	< 0.001	39 (29.1)	0.001
New IVH^c^	81 (16.2)	80 (5.3)	< 0.001	18 (13.0)	0.001
Change in hematoma volume (mL)	15.9 (26.8)	1.5 (5.9)	< 0.001	4.3 (10.8)	0.004
Change in midline shift (mm)	2.3 (4.6)	0.3 (2.7)	< 0.001	0.6 (2.9)	0.30
Change in PHE volume (mL)	13.4 (18.8)	4.3 (7.8)	< 0.001	7.9 (13.2)	< 0.001

Data are number (%) or mean (standard deviation). Statistics are *t*-Student and chi-squared tests: ^a^comparison of early neurological deterioration and no deterioration; ^b^comparison of late neurological deterioration and no deterioration. ^c^Findings of intraventricular hemorrhage on 24-h CT when the baseline CT did not show any intraventricular hemorrhage. *IVH*, intraventricular hemorrhage; *PHE*, perihematomal edema

Multivariable logistic regression analysis showed that recruitment from the UK, admission systolic blood pressure, NIHSS, prior antiplatelet therapy, previous ICH, onset-to-CT time, baseline hematoma volume, midline shift, intraventricular hemorrhage and subarachnoid extension were independently associated with END (Table 3). The strongest predictors of END were previous ICH (adjusted odds ratio [aOR] 2.62; 95%CI 1.58–4.34; *p* < 0.001), recruitment from the UK (UK, aOR 2.40, 95%CI 1.62–3.56; *p* < 0.001) and subarachnoid extension (aOR 2.06, 95%CI 1.44–2.94; *p* < 0.001). A comparison of characteristics between UK and non-UK showed that patients recruited from the UK were older, more likely to have previous stroke and intracerebral hemorrhage, lobar hematoma, intraventricular hemorrhage, larger baseline hematoma volume, shorter onset to randomization and also more likely to be issued with a DNAR order in the first week (Supplemental Table 1). For every 10 mL increase in baseline hematoma volume, the risk of END increased by 21% (aOR 1.21, 95%CI 1.12–1.32; *p* < 0.001). The predictors of LND were older age, male sex, previous intracerebral hemorrhage, higher NIHSS and larger baseline hematoma volume (Table 3).

**Table 3:** Logistic regression analyses for predictors of neurological deterioration

Variables	Early neurological deterioration		Late neurological deterioration	
	Adjusted OR (95%CI)	*p*	Adjusted OR (95%CI)	*p*
Age (years)	1.006 (0.994–1.018)	0.32	1.024 (1.005–1.043)	0.012
Sex (male)	1.09 (0.84–1.43)	0.51	1.59 (1.03–2.45)	0.037
Country (UK)	2.40 (1.62–3.56)	< 0.001	0.89 (0.55–1.43)	0.63
Premorbid modified Rankin Scale	1.07 (0.94–1.21)	0.30	1.02 (0.84–1.24)	0.82
Systolic blood pressure (mmHg)	1.008 (1.004–1.013)	< 0.001	1.003 (0.996–1.010)	0.37
National Institutes of Health Stroke Scale	1.05 (1.03–1.07)	< 0.001	1.04 (1.00–1.07)	0.043
Onset-to-CT time (hours)	0.83 (0.74–0.93)	0.001	0.98 (0.83–1.15)	0.80
Previous antiplatelet therapy	1.77 (1.33–2.37)	< 0.001	1.16 (0.73–1.84)	0.53
Previous intracerebral hemorrhage	2.62 (1.58–4.34)	< 0.001	2.98 (1.39–6.42)	0.005
Intraventricular hemorrhage	1.57 (1.19–2.07)	0.001	1.40 (0.91–2.15)	0.13
Subarachnoid extension	2.06 (1.44–2.94)	< 0.001	1.36 (0.73–2.52)	0.33
Lobar location	1.28 (0.89–1.82)	0.18	0.82 (0.45–1.49)	0.51
Hematoma volume (per 10 mL increase)	1.21 (1.12–1.32)	< 0.001	1.26 (1.10–1.42)	< 0.001
PHE volume (per 10 mL increase)^a^	1.10 (0.98–1.24)	0.095	1.06 (0.89–1.27)	0.55
Midline shift ≥ 5 mm	1.74 (1.21–2.51)	0.003	1.35 (0.77–2.37)	0.29
Leukoaraiosis	1.31 (0.99–1.73)	0.059	0.98 (0.63–1.52)	0.93
Raised leukocyte count (> 11.0 × 10^9^/L)	0.95 (0.68–1.32)	0.74	1.07 (0.65–1.76)	0.79
Glucose (mmol/L)	1.02 (0.98–1.06)	0.36	1.04 (0.97–1.11)	0.27

All variables significant on univariate analysis were entered into the models. *PHE*, perihematomal edema

^a^Refers to baseline volume. In an alternative model, day 2 PHE volume was similarly not a significant predictor of LND (aOR 1.10 per 10 mL, 95%CI 0.96–1.26; *p* = 0.17)

We further explored if a DNAR order was associated with less active care and its effect on neurological deterioration. Patients with DNAR in the first 7 days were less likely to undergo neurosurgery (13, 2.6% vs 107, 5.9%; *p* = 0.003, chi-squared test), intensive care unit admission (38, 7.5% vs 92, 10.6%; *p* = 0.036), received antihypertensive agent(s) (364, 71.9% vs 1553, 86.3%; *p* < 0.001) but had similar invasive ventilation (34, 6.7% vs 130, 7.2%; *p* = 0.72) compared with those with no DNAR order. Of the 223 patients who died in the first 7 days, 204 (91.5%) had a DNAR order issued, 18 (8.1%) had not while the status was unknown in one patient. We performed sensitivity analyses for predictors of END and LND by excluding patients with DNAR at day 2 and day 7 respectively in the logistic regression model (Supplemental Table 2). Similar to the full model (Table 3), significant predictors of END were recruitment from the UK, admission systolic blood pressure, NIHSS, prior antiplatelet therapy, previous ICH, onset-to-CT time, baseline hematoma volume, midline shift, intraventricular hemorrhage and subarachnoid extension, with the strongest being previous ICH (aOR 2.74; 95%CI 1.44–5.19; *p* = 0.002), recruitment from the UK (aOR 1.91, 95%CI 1.23–3.05; *p* = 0.004), subarachnoid extension (aOR 2.06, 95%CI 1.44–2.94; *p* < 0.001) and midline shift (aOR 1.93, 95%CI 1.19–3.13; *p* = 0.008). Older age, previous intracerebral hemorrhage, higher NIHSS and larger baseline hematoma volume predicted LND.

Tranexamic acid significantly reduced the risk of neurological deterioration within 7 days (aOR 0.79, 95%CI 0.64–0.97; *p* = 0.026) and END (aOR 0.79, 95%CI 0.63–0.99; *p* = 0.041) but not LND (Table 4). Tranexamic acid reduced the risk of hematoma expansion (aOR 0.76, 0.62–0.93; *p* = 0.008) and hematoma progression (aOR 0.71, 0.59–0.86; *p* < 0.001) but not edema growth at 24 h. There was no significant difference in reported cerebral and non-cerebral events between the treatment groups (Table 4). Sensitivity analyses excluding patients with DNAR order yielded similar results where tranexamic acid reduced the risk of neurological deterioration within 7 days, END, hematoma expansion and hematoma progression (Supplemental Table 3).

**Table 4:** Effect of tranexamic acid on neurological deterioration, serious adverse events and radiological outcomes

	Tranexamic acid	Placebo	MD/OR (95%CI)	*p*
Neurological deterioration^a^				
All ≤ 7 days	338 (29.1)	366 (31.4)	0.79 (0.64, 0.97)	0.026
Early (< 48 h)	275 (23.7)	291 (25.0)	0.79 (0.63, 0.99)	0.041
Late (48 h to 7 days)	63 (7.1)	75 (8.6)	0.76 (0.52, 1.11)	0.15
SAE with neurological deterioration ≤ 7 days^b^				
Cerebral events	274 (23.6)	304 (26.1)	0.87 (0.72, 1.06)	0.16
Non-cerebral events	33 (2.8)	39 (3.4)	0.84 (0.53, 1.35)	0.48
Radiological outcomes^a^				
Hematoma expansion^c^	265 (25.5)	305 (29.3)	0.76 (0.62, 0.93)	0.008
Hematoma progression	462 (40.1)	533 (45.9)	0.71 (0.59, 0.86)	< 0.001
PHE growth (mL)	6.8 (11.8)	6.6 (12.7)	− 0.17 (− 1.15, 0.80)	0.73

Hematoma expansion was defined as an increase in hematoma volume of > 33% or > 6 mL on follow-up scan compared with baseline. Perihematomal edema (PHE) growth is the absolute difference in PHE volume between follow-up and baseline scans. ^a^Adjusted for age, sex, country of recruitment, admission systolic blood pressure, previous antiplatelet therapy, National Institutes of Health Stroke Scale, onset-to-randomization time, intraventricular hemorrhage and baseline hematoma volume. ^b^Unadjusted. ^c^Figure differed from previously published [9] as current analysis included additional clinical scans, and radiological findings of hematoma location and presence of intraventricular hemorrhage was based on expert adjudication rather than investigator reported. *MD*, mean difference; *OR*, odds ratio; *SAE*, serious adverse event

Neurological deteriorations were reported as SAEs in 660 (89.8%) with 75 (10.2%) not reported. A full list of SAEs that resulted in neurological deterioration is given in Supplemental Table 4. Most END (499, 84.6%) was related to a cerebral SAE, with hematoma expansion (256, 43.4%) as the most common cause. Only 6.6% (*n* = 39) of END was attributed to non-cerebral SAE. A majority of LND was attributed to a cerebral SAE (79, 54.5%), but of these only 11.7% (*n* = 17) were hematoma expansion with 11.0% (*n* = 16) attributed to cerebral edema. More than one-fifth (33, 22.8%) of LND was attributed to medical complications, the highest being pneumonia (51, 6.9%). In 166 (22.6%) patients with neurological deterioration, the cause was not specified (Supplemental Table 4).

Neurological deterioration was associated with an increased risk of death at day 7 (aOR 34.27) as well as death (aOR 8.25) and death or dependency at day 90 (aOR 4.98) (Table 5). Neurological deterioration resulted in worse disability, cognition, depression and quality of life scores at day 90 compared with patients with no neurological deterioration (Table 5). Neurological deterioration increased the risk of death and dependency regardless of whether it was early (aOR 5.18, 3.72–7.22; *p* < 0.001) or late deterioration (aOR 4.06, 2.35–7.03; *p* < 0.001). Similarly, disability, cognition, depression and quality of life scores were all worse in patients with END and LND compared with patients with no deterioration (Supplemental Table 5 and 6).

**Table 5:** Effect of neurological deterioration on day 90 outcome

Outcomes	Neurological deterioration	No neurological deterioration	OR/MD 95%CI^a^	*p*
Day 7, death	214 (29.1)	9 (0.6)	34.27 (16.83, 69.80)	< 0.001
Day 90				
Death	380 (52.0)	117 (7.5)	8.25 (6.12, 11.10)	< 0.001
Modified Rankin Scale > 3	636 (87.0)	618 (39.4)	4.98 (3.70, 6.70)	< 0.001
Barthel Index	17.5 (34.7)	69.9 (37.2)	− 29.4 (− 32.4, − 26.5)	< 0.001
EuroQoL-5D Health Utility Scores	0.09 (0.27)	0.46 (0.39)	− 0.19 (− 0.23, − 0.16)	< 0.001
Telephone Interview Cognitive Status	2.1 (8.0)	20.1 (10.0)	− 10.0 (− 11.1, − 8.9)	< 0.001
Zung Depression Scale	93.6 (21.7)	53.6 (23.5)	22.1 (19.3, 24.9)	< 0.001

^a^Adjusted for age, sex, country of recruitment, previous antiplatelet therapy, National Institutes of Health Stroke Scale, admission systolic blood pressure, onset-to-randomization time, baseline hematoma volume, intraventricular hemorrhage and treatment with tranexamic acid

## Discussion

In this secondary analysis of one of the largest trials in acute ICH, significant predictors of END were recruitment from UK, higher admission SBP, higher NIHSS, previous ICH, antiplatelet therapy, shorter onset-to-CT time, intraventricular hemorrhage, subarachnoid extension, larger hematoma volume and midline shift. Older age, male sex, higher NIHSS, previous ICH and larger hematoma volume predicted LND. This is in agreement with previous findings, where patients with more severe stroke and larger hematoma were more likely to suffer from neurological deterioration [1–3, 5, 16]. Treatment with tranexamic acid was associated with reduced END but not LND.

Several predictors of neurological deterioration, i.e. baseline hematoma volume, antiplatelet therapy and shorter onset-to-CT time were also known predictors of hematoma expansion [17]. In addition, hematoma expansion was more than 3 times more common in patients with END compared with those with no neurological deterioration (58.9% vs 17.5%). Patients with no neurological deterioration had minimal change in hematoma volume (1.5 mL) at 24 h while the absolute increase in hematoma volume was significantly greater in patients with neurological deterioration (16 mL). Our results reaffirm previous findings that hematoma expansion was a main contributing factor to neurological deterioration [2–4]. Hematoma expansion was not included in the prediction model as this was not a baseline variable, hence the difficulty in establishing the temporal relation between hematoma expansion and neurological deterioration.

Intraventricular and subarachnoid hemorrhages may be associated with complications of hydrocephalus or inflammation, contributing to neurological deterioration [18]. Association of subarachnoid extension with END was similarly described in the INTERACT-2 trial and may be attributable to a higher risk of hematoma expansion [6, 16]. PHE at baseline and 24 h was not associated with neurological deterioration. Previous studies showed that larger PHE volumes did not always worsen outcome [19]. Early PHE may be a marker of clot retraction, a hemostatic process which may improve outcome later [20]. Our study did not explore the mechanisms of PHE or its evolution when it continues to grow two to three weeks after ICH [21].

Another significant finding in this study was that patients recruited from the UK had more END than those recruited outside the UK. This may be because patients recruited from the UK were older, with a higher proportion who had previous stroke, ICH, antiplatelet therapy, intraventricular hemorrhage and larger hematoma volume. As previous epidemiological studies have not indicated a higher case fatality rate of ICH in the UK [22, 23], this may reflect a trial-specific recruitment pattern where UK investigators were more likely to use brief emergency consent (29% vs 6% in non-UK). The use of full consent, which prolonged the time to randomization, may have excluded some patients with more severe stroke who may have deteriorated while consent was being sought.

DNAR order may limit active medical care, contributing to worse outcome [24] which manifest initially as neurological deterioration. However, the temporal relation between DNAR order and neurological deterioration was unclear in a large proportion of patients in this study. It is possible that DNAR orders were issued in response to neurological deterioration and may not be causal. Hence, we have not included DNAR status in the main analyses for predictors of END and LND. Excluding patients with DNAR order in the sensitivity analyses did not affect the significance of the other predictors.

In our study, tranexamic acid had a modest effect in reducing hematoma expansion at 24 h and END but not LND. This suggests that tranexamic acid may have reduced END through prevention of fibrinolysis. The role of fibrinolysis in ICH was further supported by a substudy of this trial, which showed elevated plasminogen activators in patients with hematoma growth [25]. The effect of tranexamic acid on reduction of hematoma expansion may have resulted in patients with smaller hematoma volume and less severe ICH who were less likely to suffer from complications later. Tranexamic acid, which is mostly eliminated after 24 h [26], could not have had an effect on events that occurred beyond its therapeutic window such as worsening PHE and medical complications. Hence, the early benefit of tranexamic acid was not sufficient to result in a significant reduction in LND or improved functional outcome at day 90. The recent Hemorrhagic Stroke Academia Industry (HEADS) Roundtable recommended a multimodal approach to ICH trials, combining hemostatic therapies with anti-edema agent(s) due to these reasons [27]. In addition, tranexamic acid had been shown to have some anti-inflammatory properties in clinical studies [28] and perhaps repeated dosing of tranexamic acid could reduce edema apart from promoting hemostasis.

Neurological deterioration, whether early or late, was associated with significantly higher risk of death at days 7 and 90 as well as death and dependency and worse disability, cognition, depression and quality of life scores. This highlights the need to better study, prevent and treat possible causes of neurological deterioration in clinical and research settings.

The strength of our study is the large sample size. Using an inclusive definition, we were able to determine if neurological deterioration occurred in almost all patients. However, there were difficulties in attributing a cause in one-fifth of neurological deterioration and missing follow-up scans in nearly one-fourth of participants with END. Routine neuroimaging was not performed beyond 24 h making the study of possible mechanism for LND including PHE suboptimal. As a majority of the patients were recruited from the UK, who were different compared with patients from other countries, caution should be taken in generalizing the findings. Lastly, as these were post hoc analyses, some findings may be due to chance arising from multiplicity of testing. In the analyses on the effects of tranexamic acid, we endeavoured to reduce type 1 error by including variables specified a priori in the statistical analysis plan [10].

In conclusion, neurological deterioration increased the risk of death and dependency at day 90. Tranexamic acid reduced the risk of END, probably through reduction of hematoma expansion and warrants further investigation. More studies are needed to understand the mechanisms leading to neurological deterioration in ICH.

## Electronic Supplementary Material

ESM 1 (DOCX 33 kb)

## Data Availability

The trial data can be shared, upon reasonable request to the corresponding author and trial steering committee.
