# Supplementary material for: Predictors and Outcomes of Neurological Deterioration in Intracerebral Hemorrhage: Results from the TICH-2 Randomized Controlled Trial
Source: Transl Stroke Res. 2020 Sep 9;12(2):275–83. doi: 10.1007/s12975-020-00845-6 (PMC7925446; doi:10.1007/s12975-020-00845-6)
Supplement: Supplementary file 1 — (DOCX 33 kb) [file 12975_2020_845_MOESM1_ESM.docx]

Table S1 Comparison of characteristics of patients recruited from UK and outside UK

| **Characteristics** | **UK** | **Non-UK** | **P** |
| --- | --- | --- | --- |
| Age | 69.7 (13.7) | 65.1 (13.8) | <0.001 |
| Sex, male | 1041 (54.5) | 260 (62.7) | 0.002 |
| Premorbid modified Rankin Scale | 0 [0, 1] | 0 [0, 0] | <0.001 |
| NIHSS | 12 [6, 19] | 12 [8, 18] | 0.92 |
| Prior antiplatelet therapy | 518 (27.1) | 93 (22.4) | 0.048 |
| Systolic blood pressure | 174.6 (30.6) | 175.5 (27.2) | 0.57 |
| Previous ischemic stroke/TIA | 250 (13.2) | 80 (19.4) | 0.001 |
| Previous intracerebral hemorrhage | 112 (5.9) | 14 (3.4) | 0.041 |
| Ischemic heart disease | 172 (9.1) | 31 (7.5) | 0.29 |
| Hypertension | 1105 (58.3) | 316 (76.1) | <0.001 |
| Diabetes mellitus | 251 (13.2) | 61 (14.7) | 0.41 |
| Atrial fibrillation | 53 (2.8) | 18 (4.3) | 0.096 |
| Onset-to-CT time (hours) | 2.3 (1.3) | 2.2 (1.4) | 0.32 |
| Onset-to-randomization (hours) | 3.8 (1.6) | 4.5 (1.9) | <0.001 |
| Brief consent | 558 (29.2) | 25 (6.0) | <0.001 |
| Lobar hematoma | 605 (32.2) | 83 (20.6) | <0.001 |
| Intraventricular hemorrhage | 611 (32.3) | 96 (23.8) | 0.001 |
| Hematoma volume (mL) | 25.3 (27.8) | 17.8 (22.9) | <0.001 |
| **Management within the first week** |  |  |  |
| Treated with tranexamic acid | 254 (49.9) | 207 (49.9) | 0.98 |
| Invasive ventilation | 130 (6.8) | 36 (8.7) | 0.18 |
| Intensive care | 192 (10.1) | 40 (9.7) | 0.80 |
| DNAR | 471 (24.8) | 38 (9.2) | <0.001 |
| Neurosurgery | 95 (5.0) | 26 (6.3) | 0.28 |
| **Day 90 outcome** |  |  |  |
| Modified Rankin Scale of 4-6 | 1073 (56.7) | 186 (44.8) | <0.001 |
| Death | 422 (22.3) | 77 (18.6) | 0.093 |

Data are number (%), median [interquartile range] or mean (standard deviation). Statistics are t-Student, Mann-Whitney U and Chi-squared test. DNAR=do not attempt resuscitation; NIHSS=National Institutes of Health Stroke Scale; TIA=transient ischemic attack

Table S2 Logistic regression analyses for predictors of neurological deterioration excluding patients with DNAR order

| **Variables** | **Early neurological deterioration^a^** | | **Late neurological deterioration^b^** | |
| --- | --- | --- | --- | --- |
|  | **Adjusted OR (95%CI)** | **P** | **Adjusted OR (95%CI)** | **P** |
| Age (years) | 0.997 (0.983-1.012) | 0.73 | 1.023 (1.001-1.045) | 0.043 |
| Sex (male) | 1.20 (0.895-1.68) | 0.31 | 1.32 (0.78-2.21) | 0.30 |
| Country (UK) | 1.91 (1.23-3.05) | 0.004 | 1.08 (0.61-1.92) | 0.80 |
| Premorbid modified Rankin Scale | 1.03 (0.87-1.24) | 0.71 | 0.96 (0.74-1.25) | 0.77 |
| Systolic blood pressure (mmHg) | 1.007 (1.001-1.012) | 0.017 | 1.002 (0.993-1.010) | 0.70 |
| National Institute of Health Stroke Scale | 1.04 (1.01-1.07) | 0.009 | 1.05 (1.00-1.09) | 0.033 |
| Onset-to-CT time (hours) | 0.83 (0.72-0.96) | 0.011 | 0.98 (0.81-1.19) | 0.82 |
| Previous antiplatelet therapy | 1.49 (1.01-2.20) | 0.043 | 1.17 (0.66-2.08) | 0.59 |
| Previous intracerebral hemorrhage | 2.74 (1.44-5.19) | 0.002 | 2.66 (1.04-6.80) | 0.041 |
| Intraventricular hemorrhage | 1.46 (1.02-2.08) | 0.041 | 1.67 (0.99-2.79) | 0.052 |
| Subarachnoid extension | 2.13 (1.35-3.37) | 0.001 | 0.86 (0.36-2.04) | 0.73 |
| Lobar location | 1.50 (0.95-2.35) | 0.079 | 1.08 (0.52-2.23) | 0.84 |
| Hematoma volume (per 10 mL increase) | 1.18 (1.05-1.32) | <0.001 | 1.22 (1.04-1.42) | 0.012 |
| PHE volume (per 10 mL increase) | 1.10 (0.94-1.28) | 0.095 | 0.95 (0.74-1.23) | 0.72 |
| Midline shift ≥5mm | 1.93 (1.19-3.13) | 0.008 | 2.25 (1.17-4.33) | 0.015 |
| Leukoaraiosis | 1.35 (0.95-1.94) | 0.099 | 1.00 (0.59-1.69) | 0.99 |
| Raised leukocyte count (>11.0 X10^9^/L) | 1.09 (0.72-1.66) | 0.68 | 1.43 (0.80-2.55) | 0.23 |
| Glucose (mmol/L) | 1.01 (0.96-1.07) | 0.67 | 1.03 (0.95-1.11) | 0.54 |

All variables significant on univariate analysis were entered into the models. PHE=perihematomal edema. ^a^Excluding patients with DNAR order at day 2; ^b^excluding patients with DNAR order at day 7.

Table S3 Effect of tranexamic acid on neurological deterioration, serious adverse events and radiological outcomes excluding patients with DNAR order

|  | Tranexamic acid | Placebo | MD/OR (95%CI) | p |
| --- | --- | --- | --- | --- |
| **Neurological deterioration^a^** | | | | |
| All ≤7 days | 163 (18.2) | 199 (22.0) | 0.76 (0.59, 0.99) | 0.039 |
| Early (<48 hours) | 155 (17.1) | 177 (19.8) | 0.75 (0.57, 0.98) | 0.034 |
| Late (48 hours to 7 days) | 41 (5.3) | 51 (6.7) | 0.78 (0.52, 1.19) | 0.25 |
| **SAE with neurological deterioration ≤7 days^b^** | | | | |
| Cerebral events | 147 (15.3) | 177 (18.5) | 0.80 (0.63, 1.01) | 0.062 |
| Non-cerebral events | 25 (2.6) | 28 (2.9) | 0.89 (0.51, 1.53) | 0.67 |
| **Radiological outcomes^a^** | | | | |
| Hematoma expansion | 200 (22.5) | 20 (26.0) | 0.76 (0.61, 0.96) | 0.019 |
| Haematoma progression | 314 (32.8) | 375 (39.2) | 0.69 (0.56,0.85) | 0.001 |
| PHE growth (mL) | 5.9 (9.8) | 5.7 (11.2) | -0.031 (-0.95, 0.88) | 0.95 |

Hematoma expansion was defined as an increase in hematoma volume of >33% or >6mL on follow-up scan compared to baseline. Perihematomal edema (PHE) growth is the absolute difference in PHE volume between follow-up and baseline scans. ^a^Adjusted for age, sex, country of recruitment, systolic blood pressure, previous antiplatelet therapy, National Institute of Health Stroke Scale, onset to randomisation time, intraventricular hemorrhage and baseline hematoma volume. ^b^Unadjusted. Patient with DNAR order at day 2 excluded from analyses of early neurological deterioration and radiological outcomes; patient with DNAR order at day 7 excluded from analyses of late neurological deterioration and neurological deterioration ≤7 days. MD=mean difference; OR=odds ratio; SAE=serious adverse event

Table S4 Serious adverse event classification of early and late neurological deterioration

| **Serious adverse event classification** | **END (n=590)** | **LND (n=145)** | **Total (n=735)** |
| --- | --- | --- | --- |
| Not reported | 47 (8.0) | 28 (19.3) | 75 (10.2) |
| Not serious adverse event | 5 (0.8) | 4 (2.8) | 9 (1.2) |
| Not relevant, > 7days | - | 1 (0.7) | 1 (0.1) |
| **Cerebral events** | 499 (84.6) | 79 (54.5) | 578 (78.6) |
| Expansion of haemorrhagic stroke | 256 (43.4) | 17 (11.7) | 273 (37.1) |
| Neurological deterioration (Cause not specified) | 138 (23.4) | 28 (19.3) | 166 (22.6) |
| Cerebral edema | 41(6.9) | 16 (11.0) | 57 (7.8) |
| Hydrocephalus | 29 (4.9) | 6 (4.1) | 35 (4.8) |
| Seizure | 20 (3.4) | 8 (5.8) | 28 (3.8) |
| Sedation | 6 (1.1) | 1 (0.7) | 7 (1.0) |
| Recurrent intracerebral hemorrhage | 4 (0.7) | - | 4 (0.5) |
| Ischemic stroke | 3 (0.5) | 2 (1.4) | 5 (0.7) |
| Transient ischemic attack | - | 1 (0.7) | 1 (0.1) |
| Stroke undetermined | 1 (0.2) | - | 1 (0.1) |
| Loss of consciousness | 1 (0.2) | - | 1 (0.1) |
| **Non-cerebral events** | 39 (6.6) | 33 (22.8) | 72 (9.8) |
| Pneumonia/chest infection/LRTI | 29 (4.9) | 22 (15.1) | 51 (6.9) |
| STEMI/NSTEMI | 2 (0.4) | - | 2 (0.3) |
| Pulmonary embolism | 1 (0.2) | 1 (0.7) | 2 (0.3) |
| Infection (not otherwise specified) | 1 (0.2) | 1 (0.7) | 2 (0.3) |
| Urinary tract infection | 1 (0.2) | 2 (1.4) | 3 (0.4) |
| Exacerbation of COPD | 1 (0.2) | - | 1 (0.1) |
| Fall | 1 (0.2) | - | 1 (0.1) |
| Fatigue | 1 (0.2) | - | 1 (0.1) |
| Hypotension | 1 (0.2) | - | 1 (0.1) |
| Alcohol withdrawal | 1 (0.2) | 1 (0.7) | 2 (0.3) |
| Malignancy | - | 2 (1.4) | 2 (0.3) |
| Cardiac failure/pulmonary edema | **-** | 2 (1.4) | 2 (0.3) |
| Electrolyte imbalance | **-** | 1 (0.7) | 1 (0.1) |
| Anaemia | - | 1 (0.7) | 1 (0.1) |

COPD=chronic obstructive pulmonary disease; END= early neurological deterioration; LND= late neurological deterioration; LRTI=lower respiratory tract infection; STEMI/NSTEMI=ST elevation myocardial infarction/Non-ST elevation myocardial infarction

Table S5 Effect of early neurological deterioration on day 90 outcome

| **Outcomes** | **Early Neurological deterioration** | **No neurological deterioration** | **OR/MD 95% CI^*^** | **P** |
| --- | --- | --- | --- | --- |
| Day 7, death | 196 (33.2) | 9 (0.6) | 41.97 (20.33, 86.62) | <0.001 |
| Day 90 |  |  |  |  |
| Death | 315 (53.8) | 117 (7.5) | 8.80 (6.40, 12.10) | <0.001 |
| mRS>3 | 513 (87.5) | 618 (39.4) | 5.18 (3.72, 7.22) | <0.001 |
| Barthel Index | 16.5 (34.0) | 69.9 (37.2) | -29.5 (-32.6, -26.3) | <0.001 |
| EQ5D HUS | 0.08 (0.27) | 0.46 (0.39) | -0.19 (-0.23, -0.16) | <0.001 |
| TICS | 1.9 (7.8) | 20.1 (10.0) | -9.9 (-11.1, -8.7) | <0.001 |
| ZDS | 94.1 (21.0) | 53.6 (23.5) | 22.2 (19.2, 25.2) | <0.001 |

^*^Adjusted for age, sex, country of recruitment, previous antiplatelet therapy, National Institute of Health Stroke Scale, systolic blood pressure, onset to randomization time, baseline haematoma volume, intraventricular haemorrhage and treatment with tranexamic acid. EQ5D HUS=EuroQoL-5D Health Utility Scores; MD=mean difference; mRS=modified Rankin Scale; OR= odds ratio; TICS=Telephone Interview Cognitive Status; ZDS= Zung Depression Scale

Table S6 Effect of late neurological deterioration on day 90 outcome

| Outcomes | Late neurological deterioration | No neurological deterioration | OR/MD 95% CI^*^ | P |
| --- | --- | --- | --- | --- |
| Day 7, death | 18 (12.4) | 9 (0.6) | 9.70 (3.49, 26.96) | <0.001 |
| Day 90 |  |  |  |  |
| Death | 65 (44.8) | 117 (7.5) | 6.00 (3.71, 9.70) | <0.001 |
| mRS>3 | 123 (84.8) | 618 (39.4) | 4.06 (2.35, 7.03) | <0.001 |
| Barthel Index | 21.8 (37.4) | 69.9 (37.2) | -24.8 (-30.0, -19.7) | <0.001 |
| EQ5D HUS | 0.12 (0.29) | 0.46 (0.39) | -0.15 (-0.21, -0.09) | <0.001 |
| TICS | 2.9 (8.8) | 20.1 (10.0) | -8.5 (-10.4, -6.5) | <0.001 |
| ZDS | 91.0 (24.6) | 53.6 (23.5) | 19.0 (14.0, 24.0) | <0.001 |

^*^ Adjusted for age, sex, country of recruitment, previous antiplatelet therapy, National Institute of Health Stroke Scale, systolic blood pressure, onset to randomization time, baseline haematoma volume, intraventricular haemorrhage and treatment with tranexamic acid. EQ5D HUS=EuroQoL-5D Health Utility Scores; MD=mean difference; mRS=modified Rankin Scale; OR= odds ratio; TICS=Telephone Interview Cognitive Status; ZDS= Zung Depression Scale
